# Collaborative Innovation Network, Knowledge Base, and Technological Innovation Performance-Thinking in Response to COVID-19

**DOI:** 10.3389/fpsyg.2021.648276

**Published:** 2021-09-07

**Authors:** Su Jialu, Ma Zhiqiang, Zhu Binxin, Xie Haoyang, Agyeman Fredrick Oteng, Weijun Hu

**Affiliations:** ^1^School of Management, Jiangsu University, Zhenjiang, China; ^2^Centre for Competitive Creative Design (C4D), Cranfield University, Cranfield, United Kingdom; ^3^School of Mathematical Science, Zhejiang University, Zhejiang, China; ^4^School of Archaeology, Jilin University, Changchun, China

**Keywords:** COVID-19, collaborative innovation network, network relationship strength, knowledge base breadth, technological innovation performance

## Abstract

Amid the pandemic of COVID-19, the collaborative innovation network of enterprises is conducive to the sharing of innovation resources, knowledge transfer, and technology diffusion, which is closely related to the improvement of corporate technological innovation performance. Based on the patent application data of listed enterprises in Jiangsu, Zhejiang, and Shanghai in China, this study constructs a cooperation matrix, describes the characteristics of collaborative innovation network from two dimensions of network structure and network relationship, introduces the breadth of the knowledge base as a moderating variable, and analyzes the nexus between characteristics of a collaborative innovation network and technological innovation performance. Based on the panel data of 193 listed companies in Jiangsu, Zhejiang, and Shanghai, this study uses a multiple linear regression model for empirical analysis. The results show a U-shaped relationship between clustering coefficient and technological innovation performance. The breadth of knowledge base strengthens the positive relationship between the structural hole and technological innovation performance. In contrast, the breadth of knowledge base weakens the positive relationship between network relationships strength and technological innovation performance. The study findings will enhance enterprises’ participation in a suitable collaborative innovation according to their knowledge-based characteristics and improve the technological innovation performance.

## Introduction

The outbreak and spread of COVID-19 have brought considerable challenges to our economic and social activities. It threatens the lives and health of people, and brings significant impacts on economies, finance, industries, regions, and business. According to the latest statistics data updated in the National Bureau of Statistics of China,^1^ from January to February in 2020, the overall value-added of industries above the scale experienced a significant decline of 13.5% year-on-year, in sharp contrast to the figures of 5.3% growth in the same period of 2019 (Wang and Gao, 2020). Under such a severe background, enterprises must carry out cooperative innovation to reduce transaction costs and gain a competitive advantage. In fighting the COVID-19 epidemic in China, the collaborative innovation of the entire industry has played an active role. Scientific and technological forces, innovation, and industry-university-research collaboration have played an integration and a substantial supporting role (Ke, 2020).

Collaborative innovation in response to the COVID-19 pandemic has accelerated the development of the global biomedical industry and related research fields, and pharmaceutical companies have received unprecedented attention (Sampat and Shadlen, 2021). At the same time, industry-university-research cooperation has brought them significant development opportunities. The biopharmaceutical industry has the intensification of economic globalization nowadays, but any industry’s progress and development are inseparable from collaborative innovation. Enterprises try to form collaborative innovation networks with other external enterprises. The existence of a collaborative innovation network presents many competitive advantages to the participating enterprises, which can reduce the transaction costs in Research and Development (R&D) and promote the sharing of innovation resources, knowledge transfer, and technology diffusion among enterprises and related fields. However, cooperative innovation between enterprises and universities is not always smooth. Inventors in firms face a large burden given their limited involvement with academia (Murray, 2002; Agrawal and Henderson, 2009). In other words, it is difficult for firms to choose suitable cooperative partners. The problem of selecting suitable joint partners raises a key question regarding the cooperation between academia and industry: What kind of collaborative innovation network can facilitate the flow of knowledge from academia to the industry that will drive and improve the technological innovation performance of enterprises? In addition, what role do the knowledge base play between the characteristics of a collaborative innovation network and enterprise technological innovation performance?

In the process of cooperation, not only the network characteristics can affect the innovation performance of enterprises, but also the nature of knowledge recombination in firms is vital to innovation (Bikard and Marx, 2020). In an environment of effective and efficient cooperation, enterprises with a rich knowledge base are more likely to succeed in the process of collaborative innovation. In such circumstances, enterprises are more likely to recombine knowledge (Speldekamp et al., 2020). More and more enterprises are committed to cooperative innovation to adapt to the new environment during global pandemic, thus reducing environmental pollution (Sarfraz et al., 2020a). Building and participating in the appropriate collaborative innovation network based on enterprises’ knowledge base to improve their innovation performance are a major issue worthy of attention in academia. However, existing researches pay more attention to the direct impact of network structure characteristics or enterprises’ knowledge base on innovation performance, while most of them focus on the industrial level. This study employs the data of listed enterprises in Jiangsu, Zhejiang, and Shanghai and discusses the logical relationship and characteristics between collaborative innovation networks and technological innovation performance. This study includes the two dimensions of network structure and network relationship. The breadth of enterprises’ knowledge base is added as a moderating variable to study its direct impact on technological innovation performance and its moderating role between various network characteristics and technological innovation performance. From the practical level, this study provides important management enlightenment for middle- and high-level managers, local governments, and research institutions of Chinese enterprises on how to improve innovation ability and achieve win-win cooperation. From the theoretical level, supported by social network theory, resource-based theory, and collaborative innovation-related theories, the researchers have expanded the related studies on collaborative innovation networks, enterprise cooperation networks, etc. This study contributes significantly to previous studies in terms of index measurement and theoretical model construction.

The study is organized as follows: the researchers summarize the related literature and put forward hypotheses in Theoretical Development And Hypothesis Development. We introduce the sample data and describe the variables in Sample Data and Variable Description. The empirical results and analysis are presented in Empirical Results and Analysis. We offer a discussion of the policy implications of our results and concludes the paper in Discussion and Conclusion.

## Theoretical Development and Hypothesis Development

Granovetter (1985) divided network features into network structure and network relationship from embeddedness regarding the division of collaborative innovation network dimensions. He pointed out that the network relationship mainly refers to the content, direction, and strength of relationships. In contrast, the structure refers to the distribution of network relationships in the overall network, including location, scale, and density. These can all be the specific measurement indexes of network characteristics. Extant studies indicate that many scholars have independently studied the impact of network structure and network relationships on corporate innovation performance. However, few studies have combined the two dimensions of structure and relationship to explore the relationship between network characteristics and corporate innovation performance. In addition, with the increasingly frequent sharing and transfer of knowledge resources between different subjects, the scope of knowledge-based theories has gradually expanded, not limited to a single enterprise, which also provides a theoretical basis for collaborative innovation network research amid the COVID-19 pandemic period. Based on this theoretical development, this article put forward relevant hypotheses to support the findings.

### The Impact of Network Structure on Technological Innovation Performance of Enterprises

Firstly, the network clustering coefficient has an enormous impact on the technological innovation performance of enterprises. A higher degree of agglomeration can enhance the trust relationship between partners in a cooperative network with small-world characteristics. The network clustering coefficient may conduct more frequent interaction and cooperation, which effectively improve the efficiency and accuracy of communication while reducing the risk of information dissemination, minimizing the innovation cost of enterprises, and finally increasing the innovation output of enterprises (Xia et al., 2018; Zhang et al., 2020). However, the innovation network with a high clustering coefficient will make the information aspect homogeneous, resulting in the phenomenon of local information circulation and resource redundancy, which will damage the efficiency of the network. The damage of the efficiency of the network inhibits the innovation output of enterprises and preventing the entrance of new partners (Uzzi and Spiro, 2005; Jiang et al., 2018). Hui and Xiaomin (2017) used the data of the Shanghai pharmaceutical manufacturing industry as samples to explore the influence of network characteristics on enterprise innovation performance. The result indicated a negative correlation between the network’s small-world characteristics (average path length and clustering coefficient) and enterprise innovation performance. Jieqiong et al. (2015) believed that at the initial stage of constructing a cooperative network, the main body in the network has less experience in cooperation and gradually enters the formation stage of cooperation practice and ability. During this period, the pattern of cooperation is highly uncertain, and the clustering coefficient negatively affects enterprise innovation performance. However, when the cooperation experience of the main body is gradually enriched and the cooperation network begins to take shape, the promotion of the clustering coefficient can also promote the innovation performance of enterprises.

Based on the above analysis, this study argues that at the inception of a collaborative innovation network, the subjects’ lack of technical cooperation experience leads to a low degree of cooperation and coordination. Thus, there are few information resources in the network. The increase of the clustering coefficient is not conducive to information flow, which hinders the improvement of the technological innovation performance of enterprises. However, with the gradual increase of network scale, enterprises’ accumulated innovation resources and cooperation experience increase daily. Therefore, it becomes convenient to exchange knowledge resources and to cooperate accurately and efficiently. The higher the degree of network aggregation, the easier it is to promote the technological innovation performance of enterprises. The following hypothesis is put forward:

H1a: *There is a U-shaped relationship between the clustering coefficient and technological innovation performance of enterprises*.

Secondly, the numbers of structural holes have an enormous impact on the technological innovation performance of enterprises.

Nodes with more structural holes often have greater power, especially control over the flow of resources in the network (Yan and Guan, 2018). Therefore, firms that occupy a higher number of structural holes are often considered to be focal firms, and they always benefit by being better informed about what is going on in the network (Macaulay et al., 2018; Watts and Koput, 2019) and by bringing in high-quality resources (Sullivan et al., 2007). Coleman (1998) put forward a critical point of view on the relationship between structural holes and enterprise innovation performance. He pointed out that enterprises with more structural holes can learn the latest market situation faster and adjust their innovation strategy to adapt to the new changes in the market. It also promotes the improvement of enterprise innovation performance. Zaheer and Bell (2005) found that enterprises in the intermediary position have more innovation opportunities to keep their competitive advantage all the time. Jianzhong and Yingying (2015)also found that structural holes significantly positively impact the enterprise innovation performance.

Further, Karamanos (2016) suggested that alliance partners’ structural holes positively affect the focal firms’ exploratory innovation. Zhang et al. (2020) found that a greater betweenness centrality means more structural holes can facilitate the transmission of complementary knowledge. Wang and Sun (2021) showed that structural holes have a positive impact on green technology innovation. However, Liu (2011) pointed out that when the overall network is sparse, the number of structural holes does not positively impact enterprise innovation performance. Chenlei et al. (2017) showed that structural holes are negatively correlated with enterprise innovation performance.

This study argues that enterprises with more structural holes can get more cooperation opportunities. In this direction, if the enterprises can accurately identify the innovative resources that are beneficial and use them, the technological innovation performance of the enterprises can be significantly improved. Thus, it is hypothesized that:

H1b: *There is a positive relationship between structural holes and the technological innovation performance of enterprises*.

### The Impact of Network Relationship on Technological Innovation Performance of Enterprises

Network relationship strength refers to the connection strength between two network members. Ritter and Gemünden (2003) pointed out that when an enterprise has close ties with external subjects, it is possible to obtain a higher success rate of product and process innovation. Hongming et al. (2012) found that the intensity of network relationships directly impacts the enterprises’ technological innovation. An empirical study by Jianping and Xiaoyun (2019) showed that the intensity of network relationships has a positive effect on improving the innovation performance of enterprises. The high intensity of network relationships has driven the rapid development of the industry while driving companies’ technological innovation. According to Sina Pharma’s statistics, in the biopharmaceutical industry,^2^ a total of 139 deals took place in the first 4months of 2020 regarding R&D collaborations on COVID-19 drugs, with 55% of these deals being non-government led and funded mainly by corporate collaborations. However, some scholars have a different view; that is, there are many strong links in the network, which will hinder the growth of the whole network innovation standards. Just as people usually find strong connections in close social circles, the influence of these strong connections may always remain in the network, thus inhibiting the growth of innovation (Onnela et al., 2007).

From the researchers’ perspective, the enterprise collaborative innovation network emphasized that the strong relationship of enterprises has a higher degree of trust, which plays a positive role in reducing opportunistic threats, contributes to the resource sharing and further in-depth cooperation between the two partners, and can effectively improve the technological innovation performance of enterprises. Based on this, it is postulated that:

H2: *There is a positive relationship between the intensity of network relationships and the technological innovation performance of enterprises*.

### The Direct Role and Regulatory Role of the Breadth of the Knowledge Base

1. The relationship between the breadth of knowledge base and technological innovation performance of enterprises. The breadth of knowledge base refers to the types of all knowledge elements owned by enterprises. In terms of enterprises, the more types there are, the wider technical fields there are. Studies have shown that if an enterprise can master knowledge in multiple technical fields, its absorptive capacity will be enhanced. In other words, the breadth of the enterprise knowledge base determines its ability to evaluate the scope of external knowledge (Cohen and Levinthal, 1990). Yan and Hong (2011) found that the breadth of knowledge base positively promotes the technological innovation performance of enterprises and formation of technological cooperation relations among enterprises (Hong et al., 2013). An empirical study by Chaoying and Lina (2017) showed that the diversity of enterprise knowledge base had a significant positive effect on knowledge innovation.
  This study argues that the broader the knowledge base of enterprises, the stronger their absorptive capacity. They can learn more about the cooperative innovation network and then effectively improve their technological innovation performance. Based on this, the following assumption is put forward:
  H3: *The breadth of the knowledge base is positively related to the technological innovation performance of enterprises*.
2. The adjustment of the breadth of knowledge base to the relationship between clustering coefficient and technological innovation performance of enterprises.
  Laursen and Salter (2006) indicated that extensive and in-depth search through various search channels could provide ideas and resources that are helpful for enterprises to obtain and utilize innovation opportunities. However, with increased enterprise knowledge elements, the barriers to knowledge exchange between departments increase, inhibiting innovation performance improvement. Therefore, in a cooperative innovation network with a high degree of aggregation, frequent interaction between innovation subjects is beneficial to increase the knowledge base of enterprises. However, enterprises need to spend more time and energy to maintain and develop new knowledge elements to accumulate knowledge elements. At the same time, communication barriers between departments increase, resulting in a decrease in innovation output. Han and Yan (2017) took 171 enterprises in the information technology industry as research objects. The empirical results showed that the knowledge breadth had a negative moderating effect on the relationship between network clustering coefficient and enterprise innovation (number of granted patents). Based on this, the following assumption is put forward:
  H4a: *The breadth of knowledge base negatively regulates the relationship between clustering coefficient and technological innovation performance of enterprises*.
3. The adjustment of knowledge breadth to the relationship between structural holes and technological innovation performance of enterprises.
  Enterprises with more structural holes often have the advantage of resource control and can obtain abundant information resources. The knowledge breadth plays a vital role in the absorptive capacity of enterprises. Suppose enterprises with a wide knowledge base occupy the advantage of structural holes at the same time. In that case, they can screen out the ideas and knowledge in different markets quickly and accurately. This is conducive to opening up the connection channels between various knowledge elements and cooperation networks within enterprises, thus optimizing the allocation of knowledge resources within enterprises, improving utilization efficiency, and contributing to innovative achievements (Zhiming, 2016; Jiang et al., 2018). Hierarchical CEO succession is conducive to the improvement of SMEs performance through effective allocation of knowledge resources (Sarfraz et al., 2020b) and has positive impact on the innovation environment within the enterprise (Sarfraz et al., 2020c). On the contrary, if an enterprise has a narrow knowledge base and a large number of structural holes, it will be able to not only absorb the rich information resources in the network but also waste a lot of human and material resources of the enterprise. This inhibits the improvement of technological innovation performance because of blind technical cooperation. Based on this, this study puts forward the following assumption:
  H4b: *The knowledge breadth positively regulates the relationship between structural holes and the technological innovation performance of enterprises*.
4. The adjustment of the breadth of knowledge base to the relationship between network relationship strength and enterprise technological innovation performance.
  Zhou and Li (2012) found that companies with an extensive knowledge base were more capable of developing fundamental innovation under the condition of internal knowledge sharing rather than external market knowledge acquisition. Yan et al. (2014) took the patent data of China’s electronic information industry as research samples to explore the role of the knowledge base (including the breadth of knowledge base and consistency of knowledge base) between enterprises’ technological cooperation and technological innovation performance. The study findings showed that there was a negative moderating effect. Therefore, enterprises can learn and absorb new knowledge stably for a long time and increase innovation output by deepening the intensity of network relations. However, when the enterprise’s knowledge base is wide, the increase of network relationship strength does not necessarily promote the improvement of technological innovation performance. First, enterprises with a wide knowledge base may be too scattered in the technical fields of cooperation with external subjects because of their wide involvement. Increasing the intensity of network relations will lead to the inability to concentrate and consume vast amounts of workforce and materials but will not benefit enterprise innovation. Secondly, the communication barriers between technical departments of enterprises will increase with the widening of technical fields, and the cost of knowledge conversion will also increase. However, the high network relationship strength makes it impossible for enterprises to give up their existing partners easily; thus, the increased knowledge conversion cost and relationship maintenance cost are not conducive to enterprise performance improvement. Based on this, this study puts forward the following assumption:
  H4c: *The breadth of knowledge base negatively regulates the relationship between the strength of network relationships and the technological innovation performance of enterprises*.

## Sample Data and Variable Description

### Sample Data

The samples used in this study are from the selected listed companies in Jiangsu, Zhejiang, and Shanghai in China. The intellectual property rights in Jiangsu, Zhejiang, and Shanghai are intensive, one of the regions with active innovation in China. The three places jointly build intellectual property protection policies to promote the integrated development of intellectual property rights. In this context, enterprises have high requirements for knowledge innovation. Enterprises begin to improve their competitive position through collaborative innovation due to increasingly fierce market competition.

In this study, the patent data used as research samples were jointly gathered from the A-share listed companies in Jiangsu, Zhejiang, and Shanghai, and other innovative subjects. The periods used were nearly 7years. Since the study period started in 2010, to ensure the availability of relevant index data, 502 listed companies in operation before 2010 were selected as research objects. The patent applications data of each listed company were initially searched independently. After the listed companies without cooperative patents were excluded, then, based on listed companies’ average annual cooperative patents, excluding the extreme values other than plus or minus three standard deviations, 193 A-share listed companies in Jiangsu, Zhejiang, and Shanghai were selected as the research objects. They comprised 58 listed companies in Jiangsu, 77 in Zhejiang, and 58 in Shanghai. The data of 193 listed companies applying for invention patents and utility model patents in China Patent Information Center (CNPAT^3^) were collected, and 7,563 remaining cooperative patents were also collected.

Most importantly, a patent cooperation matrix and the relevant network indicators are followed based on 7,511 cooperative patents after the patent data of duplicate applications were continuously eliminated. Furthermore, the design patents are not included in the research samples because of their low technical content and simple application process, which have little effect on reflecting the true technical level of enterprises. Besides, the time lag effect of R&D output is considered, and there is a lag period when the technological innovation performance of enterprises is measured. Patent data in this study are from Baiten.com,^4^ financial data, and R&D data are from the annual reports of listed companies in Juchao.com.^5^

### Variable Description

Firstly, the explained variable is the technological innovation performance of enterprises. In this study, data envelopment analysis (DEA) and MAXDEA software were used to measure the enterprises’ technological innovation performance. On the part of regression analysis, the BCC model based on variable returns to scale is selected to measure the technological innovation performance of enterprises. In the robustness test, the SBM super-efficiency model is selected to measure the dependent variable. Concerning the selection of input–output indicators, the principles of objectiveness, systematicness, representativeness, and operability of the evaluation index system are followed. At the same time, the reliability of data sources and the feasibility of data collection are considered. R&D expenses and R&D personnel are selected as input indicators. The number of patent applications and the ratio of intangible assets to total assets are selected as output indicators. Intangible assets usually include patent rights, trademark rights, copyright, franchise, secret production method, formula, etc. The proportion of intangible assets can measure the technological innovation ability of enterprises (Hongtao and Xingzhuo, 2013). Considering that there is a specific time lag in applying enterprise innovation resources, the input index of this study is the enterprise-related data in the current year, and the output index is the enterprise-related data 1year behind.

Secondly, based on the two dimensions of network structure and network relationship, the clustering coefficient, structural hole, and network relationship strength are selected as explanatory variables. The clustering coefficient is an index of the ego-network structure, which represents the tightness of the ego-network. A structural hole is used to express the non-redundant connection. The most commonly used measures of the structural hole are limitation and effective scale. Because the restriction degree is a negative index and the effective scale is a positive index, this study uses the index of effective scale to measure the structural hole. The above two indexes are measured by UCINET software. According to Xinyue et al., 2018, the strength of the network relationship refers to measuring the average strength of all direct cooperative relationships of sample enterprises. The average value of each connection is directly connected with the enterprise in the collaborative innovation network.

Again, knowledge breadth is considered as the adjustment variable for this study. The breadth of knowledge base refers to all kinds of knowledge elements owned or controlled by an enterprise. This study referred to (Hong et al., 2013) measurement method of knowledge breadth and measures its knowledge breadth by the number of major categories of all international technology classifications involved in the application for invention patents in the first 5years, that is, the number of non-duplicate International Patent Classification (IPC) numbers.

Finally, the control variables of this study are enumerated as follows:

- The age of listing is the difference between the date of listing and the data collection date. Generally speaking, enterprises listed earlier are well-known in the market. They always have stronger capital strength, richer material, human resources, and relatively complete R&D teams and equipment, making it easier to obtain more innovative output from innovation investment.
- The enterprise scale consists of the total assets at the end of each year. Generally speaking, the larger the scale of an enterprise, the stronger the financial strength, the more advanced the technology and equipment possessed, and the number of employees in the enterprise to a certain extent also took a big advantage; thus, the higher the absorption rate and utilization rate of innovative resources, the better the technological innovation performance.
- The degree of centrality, that is, the number of partners directly connected with the enterprise. Enterprises with a high degree of centrality can obtain more innovation resources to promote the improvement of the technological innovation performance of enterprises. Also, such enterprises often have a better reputation in their industries to more easily win partners’ trust and attract more partners.
- The network centrality, that is, the overall centrality of the network. Wang et al.’s (2019) research showed that enterprise network centrality had a negative impact on exploratory innovation performance. Therefore, this study considers it as one of the control variables.
- R&D investment consists of the annual expenses the enterprises incurred in relation to R&D. Most scholars believe that R&D investment plays a positive role in improving the technological innovation performance of enterprises. However, a few scholars demonstrate an inverted U-shaped relationship between R&D investment and enterprise innovation performance and assert that over-emphasizing the high intensity of enterprise innovation investment is not conducive to the improvement of enterprise innovation performance (Zhiyong, 2013). Therefore, this study takes R&D investment as one of the control variables.

To sum up, this study investigates the technological innovation performance of enterprises. The clustering coefficient, structural hole, and network relationship strength were considered as explanatory variables. The breadth of knowledge base as moderating variable specifically analyzes the relationship between collaborative innovation network characteristics, knowledge breadth, and technological innovation performance of listed enterprises in Jiangsu, Zhejiang, and Shanghai. The variables described are shown in Table 1.

**Table 1:** Description of variables.

Variable type	Name	Symbol	Measure index
Interpreted variable	Technological innovation performance	C	The efficiency of technological innovation
Explanatory variable	Clustering coefficient	CC	Network clustering coefficient
	Structural hole	SH	Effective scale
	Network relationship strength	RE	The average value of the values of each connection directly connected to the node
Control variable	Listing age	time	Number of listed years
	Enterprise scale	firm size	LN (total assets at the end of the period)
	Point degree center degree	DC	Relative pointwise centrality
	Network-centric potential	NC	Network-centric potential
	R&D investment	INV	LN (total R&D investment of enterprises)
Regulated variable	The breadth of knowledge base	KW	Number of non-duplicate IPC classification numbers

## Empirical Results and Analysis

### Descriptive Statistical Analysis

In this section, we use SPSS software to complete the descriptive statistics and regression analysis. Table 2 shows the mean deviation, standard deviation, and correlation coefficient of all involved variables. It can be observed from Table 2 that the average value of technological innovation performance is 0.7381, and the standard deviation is 0.1203, indicating that the technological innovation performance of most enterprises is close to the overall average value. The average value of structural hole, network relationship strength, and knowledge breadth is minimal. The standard deviation is large, which indicates that the variable value is relatively discrete. There are great differences among enterprises in terms of patent cooperation and knowledge base.

**Table 2:** Descriptive statistics and correlation coefficient.

	1	2	3	4	5	6	7	8	9	10
1. C	1									
2. Time	−0.234^***^	1								
3. Firm size	−0.386^***^	0.441^***^	1							
4.DC	−0.013	0.039	0.119^**^	1						
5.NC	−0.210^***^	0.186^***^	0.143^***^	−0.004	1					
6.INV	−0.566^***^	0.298^***^	0.617^***^	0.078^**^	0.209^***^	1				
7.CC	−0.303^***^	0.224^***^	0.159^***^	−0.103^**^	0.080^**^	0.160^***^	1			
8.SH	0.004	0.140^***^	0.406^***^	0.406^***^	0.008	0.292^***^	0.014	1		
9.RE	−0.032	0.090^**^	0.019	0.564^***^	0.038	0.007	0.114^**^	−0.048	1	
10.KW	−0.085^**^	0.249^***^	0.517^***^	0.226^***^	0.091^**^	0.437^**^	0.101^**^	0.601^***^	0.014	1
Mean	0.738	10.499	22.537	0.080	0.872	9.455	0.686	2.319	5.272	7.617
SD	0.120	5.768	1.280	0.138	0.372	1.706	0.036	3.227	8.897	7.807

^*^Correlation is significant at the 0.1 level (two-tailed);

** Correlation is significant at the 0.05 level (two-tailed);

*** Correlation is significant at the 0.01 level (two-tailed).

Correlation analysis was performed to determine the strength of the linear relationship between two variables. This study preliminarily examines the linear relationship between variables by measuring the correlation coefficient. It can be observed from Table 2 that the clustering coefficient, network relationship strength, and technological innovation performance of enterprises in the network have a negative correlation. In contrast, the structural hole has a positive correlation. The knowledge breadth positively affects the clustering coefficient and structural hole network relationship strength while negatively affecting technological innovation performance. However, the correlation analysis does not consider the mutual influence among multiple variables. As far as the correlation coefficient is concerned, it may not be the true embodiment of the linear correlation. Therefore, to clarify the relationship between variables, further regression analysis is needed.

### Regression Analysis

Before the regression analysis is done, all the variables involved in the interaction should be mean-centered to avoid the potential collinearity problem. Followed by hierarchical regression analysis, all regression results are shown in Table 3. It can be seen from each table that the F-statistics of all regression models have passed the significance test at 1%, which proves that the model has a good fitting degree. The linear regression model can describe and reflect the relationship among independent variables, regulating variables, and dependent variables.

**Table 3:** Results of hierarchical regression analysis.

Variable	Model 1	Model 2	Model 3	Model 4	Model 5	Model 6	Model 7
Time	−0.050	−0.009	0.005	−0.001	−0.002	0.009	0.006
Firm size	−0.039	−0.115^***^	−0.114^***^	−0.153^***^	−0.152^***^	−0.137^***^	−0.155^***^
DC	0.033	−0.150^***^	−0.138^***^	−0.132^***^	−0.130^***^	−0.078^*^	−0.140^***^
NC	−0.089^***^	−0.074^**^	−0.097^***^	−0.100^***^	−0.099^***^	−0.100^***^	−0.100^***^
INV	−0.511^***^	−0.509^***^	−0.479^***^	−0.507^***^	−0.503^***^	−0.486^***^	−0.510^***^
CC		−0.226^***^	−0.567^***^	−0.567^***^	−0.569^***^	−0.562^***^	−0.567^***^
SH		0.270^***^	0.253^***^	0.166^***^	0.174^***^	−0.051	0.163^***^
RE		0.101^**^	0.091^**^	0.083^**^	0.083^**^	0.049	0.089^**^
CC2			0.385^***^	0.381^***^	0.382^***^	0.391^***^	0.378^***^
KW				0.182^***^	0.205^***^	0.139^***^	0.167^***^
KW*CC					0.081		
KW*CC2					−0.071		
KW*SH						0.242^***^	
KW*RE							−0.058^*^
R2	0.335	0.419	0.452	0.470	0.471	0.483	0.472
Adj-R2	0.330	0.412	0.445	0.462	0.462	0.475	0.464
F	68.82^***^	61.33^***^	62.30^***^	60.11^***^	50.25^***^	57.65^***^	55.21^***^

* *Correlation is significant at the 0.1 level (two-tailed)*;

** *Correlation is significant at the 0.05 level (two-tailed)*;

*** *Correlation is significant at the 0.01 level (two-tailed)*.

Model 1 contains only control variables. From Table 3, it can be seen that listing age (*time*), enterprise scale (*firm size*), and point degree center degree (*DC*) have no significant influence on the technological innovation performance of enterprises (*p*-values are 0.158, 0.354, and 0.299, respectively). The network-centric potential (*NC*) negatively affects the technological innovation performance of enterprises, and it has passed the 1% significance level test. The reason is that the overall network-centric potential is low. The small groups in the network are relatively independent, which hinders the knowledge flow of the whole network, thus inhibiting the improvement of the technological innovation performance of enterprises. There is a negative relationship between R&D investment and technological innovation performance, and it has passed the 1% significance level test. Observing the technological innovation performance of enterprises, we can see that the overall average value is 0.738, which shows that most enterprises have redundancy in R&D investment. Listed companies are often more abundant in the capital market, and they have more R&D investment than non-listed companies. However, emphasizing the high intensity of R&D investment is not conducive to improving enterprise innovation performance (Kang, 2013). Felix Ayadi et al. (1996) thought that excessive R&D of enterprises reduced the return on investment, thus inhibiting innovation performance improvement. Therefore, the innovation activities of enterprises should follow the strategy of comparative advantage, not blindly invest in R&D, but promote technological innovation performance by improving their capabilities.

After adding independent variables in Model 2, R^2^=0.419, which is significantly higher than 0.335 in Model 1, and *F*=61.330, which has passed the 1% significance level test. Therefore, this result indicates that the influence of control variables can be eliminated, and independent variables have a strong explanatory effect on the technological innovation performance of enterprises. Table 3 shows that the standardized coefficient value of the first term of the clustering coefficient is −0.226, which passes the 1% significance level test. Therefore, the clustering coefficient has a significant negative impact on technological innovation performance. To explore whether there is a curvilinear relationship between the clustering coefficient and the technological innovation performance of enterprises, this study adds the square variable of the clustering coefficient in Model 3. The regression coefficient of the quadratic term is 0.385, which is positive at the significance level of *p*<0.01, indicating that the clustering coefficient in the patent cooperation network of listed enterprises presents a U-shaped relationship with technological innovation performance. That is to say, when the clustering coefficient in the network gradually increases, the technological innovation performance of enterprises first drops and then rises, which is consistent with the conclusion drawn by Wu and Wang (2018). And at this stage, the technological innovation performance of enterprises is decreasing with the clustering coefficient. The reason is that the construction of a collaborative innovation system of listed enterprises in China is at the initial stage, and there is still a lack of cooperation experience among enterprises, universities, and research institutes, which needs a long time to adapt. Once the cooperation subjects gather continuously, the frequency of knowledge exchange increases and the increase of clustering coefficient will promote the technological innovation performance of enterprises.

The regression coefficient of the structural hole is 0.270, which has passed the 1% significance level test. It shows a positive effect between the structural hole and technological innovation performance of enterprises. Thus hypothesis H1b is verified. In the collaborative innovation network, the formation of structural holes is conducive to enhancing the trust among enterprises. Enterprises occupying a large number of structural holes can learn more knowledge and acquire richer heterogeneous resources from the collaborative innovation network. Collaborative innovation networks can help discover new market opportunities and grasp new market conditions at the first time, promote their innovation to adapt to the new environment and improve their technological innovation performance. The regression coefficient of network relationship strength is 0.101, the *p*-value is 0.011 (less than 0.05), and the hypothesis H2 is supported. In this study, the index of network relationship strength is measured by the average patent cooperation times of enterprises. The higher the network relationship strength, the more patent cooperation times between enterprises and external innovation subjects, which also means that the higher level of trust and knowledge matches them. This is more conducive for enterprises to access mutual learning and imitate effective communication and information transmission. Thus reducing the cooperation risk brought by uncertainty and directly improving the technological innovation performance of enterprises.

Model 4 adds moderating variables based on Model 3, and it tests the direct effect of the knowledge breadth on technological innovation performance. From Table 3, it can be seen that the regression coefficient is 0.182, and the *p*-value is 0.000, which has passed the 1% significance level test. It shows a significant positive correlation between enterprises’ knowledge breadth and technological innovation performance; hence, hypothesis H3 passes the verification.

In Model 5, the product of the knowledge breadth and the first term of clustering coefficient are added and the product of the knowledge breadth and the second term of the clustering coefficient. It can be observed from Table 3 that the coefficients of the two product terms are 0.081 and −0.071, respectively, and the *p*-values are both greater than 0.1, which failed the significance test. Therefore, hypothesis H4a has not been verified. From the above analysis, the results portray that constructing a collaborative innovation system of listed enterprises in Jiangsu, Zhejiang, and Shanghai has not reached the maturity stage. There is still a lack of cooperation experience among partners. Due to inadequate knowledge mobility in the network during this adaptation period, the enterprise’s knowledge breadth has not played a regulatory role between clustering coefficient and technological innovation performance.

In Model 6, the product term of the knowledge breadth and the structural hole is added. It can be observed from Table 3 that the coefficient of the product term of the knowledge breadth and structure hole is 0.242, and the value of p is 0.000, which has passed the 1% significance level test. It portrays that the enterprise’s knowledge breadth has a positive moderating effect on the relationship between enterprises’ structural hole and technological innovation performance. The hypothesis H4b is verified accordingly. Therefore, when the knowledge base of enterprises is wide, the impact of structural holes on technological innovation performance is more significant.

Model 7 examines the moderating role of knowledge breadth in the influence of network relationship strength on the technological innovation performance of enterprises. It can be seen from Table 3; the coefficient of the product term of regulatory variables and network relationship strength is −0.058, which has passed the 10% significance level test. It shows that the breadth of knowledge base weakens the positive impact of network relationship strength on technological innovation performance; hence, hypothesis H5 is verified. This is consistent with the research conclusion of Yan et al. (2014). The wider the technical field covered by the enterprise knowledge base, the more difficult for them to integrate it. In brief, it is difficult for the new knowledge obtained from outside to be transformed into the enterprises’ innovation output. Strengthening the network relationship strength will cause enterprises to consume more human and material resources. The internal innovation resources cannot be concentrated; it will inhibit the improvement of technological innovation performance.

### Robustness Test

To improve the validity of this study, a variable replacement method is used to test the robustness of the above regression model. In the part of regression analysis, the input-oriented BCC model is used to measure the technological innovation performance of the enterprise. The SBM super-efficiency model will be used to measure it and re-estimate the original regression model in the robustness test. It can be seen from Table 4 that the F-statistics of all regression models pass the 1% significance test, indicating that the model has a good fitting degree.

**Table 4:** Robustness test.

Variable	Model 8	Model 9	Model 10	Model 11	Model 12	Model 13	Model 14
Time	−0.031	0.011	0.030	0.024	0.023	0.034	0.033
Firm size	−0.035	−0.113^***^	−0.112^***^	−0.146^***^	−0.145^***^	−0.132^***^	−0.148^***^
DC	0.047	−0.119^***^	−0.102^***^	−0.097^***^	−0.093^**^	−0.048	−0.106^**^
NC	−0.091^***^	−0.077^**^	−0.109^***^	−0.112^***^	−0.110^***^	−0.112^***^	−0.112^***^
INV	−0.496^***^	−0.495^***^	−0.451^***^	−0.476^***^	−0.472^***^	−0.458^***^	−0.481^***^
CC		−0.216^***^	−0.702^***^	−0.702^***^	−0.703^***^	−0.698^***^	−0.702^***^
SH		0.268^***^	0.244^***^	0.166^***^	0.175^***^	−0.027	0.162^***^
RE		0.076^*^	0.062	0.055	0.054	0.024	0.062
CC^2^			0.550^***^	0.546^***^	0.544^***^	0.555^***^	0.543^***^
KW				0.163^***^	0.167^***^	0.124^***^	0.145^***^
KW*CC					0.069		
KW*CC^2^					−0.038		
KW*SH						0.216^***^	
KW*RE							−0.070^**^
R^2^	0.307	0.389	0.456	0.470	0.472	0.481	0.475
Adj-R^2^	0.302	0.382	0.449	0.463	0.463	0.473	0.466
F	60.58^***^	54.15^***^	63.42^***^	60.31^***^	50.47^***^	57.20^***^	55.67^***^

* *Correlation is significant at the 0.1 level (two-tailed)*;

** *Correlation is significant at the 0.05 level (two-tailed)*;

*** *Correlation is significant at the 0.01 level (two-tailed)*.

It can be observed from Table 4; the regression coefficient of the structural hole is 0.268, which passes the 1% significance level test; and the hypothesis H1b is valid and passes the robustness test. Similarly, hypothesis H2 passes the robustness test too. The regression coefficient of the quadratic term of the clustering coefficient is 0.550, and the *p*-value is 0.000 (less than 0.1), which indicates that the clustering coefficient has a U-shaped relationship with the technological innovation performance of enterprises; the hypothesis H1a still passes the robustness test.

Based on Model 10, Model 11 adds the moderating variable. It can be seen from Table 4 that hypothesis H3 passes the robustness test. Models 12 to 14 are robust tests of the moderating effect of knowledge breadth. Based on Model 12, it is observed that the coefficients of the first term and the second term of the knowledge breadth and the clustering coefficient are 0.069 and −0.038, respectively, consistent with the previous conclusions. According to Model 13, it can be found that hypothesis H4b passes the robustness test. Model 14 verifies the moderating effect of the knowledge breadth between network relationship strength and technological innovation performance. The regression coefficient of the product term is −0.07, which has passed the 5% significance level test. Thus hypothesis H4c passes the robustness test.

To sum up, each hypothesis test is consistent with the original regression model, which shows that the linear multiple regression model established in this study is robust. The empirical analysis results are summarized as shown in Table 5.

**Table 5:** Hypothesis results.

Hypothesis	Hypothetical content	Passed situation
H1a	There is a U-shaped relationship between network clustering coefficient and technological innovation performance of enterprises; that is, with the increase of network clustering coefficient, technological innovation performance first drops and then rises	Pass
H1b	There is a positive relationship between network structure hole and technological innovation performance of enterprises	Pass
H2	There is a positive relationship between the intensity of network relationship and the technological innovation performance of enterprises	Pass
H3	The breadth of knowledge base has a significant positive impact on the technological innovation performance of enterprises	Pass
H4a	The breadth of knowledge base weakens the U-shaped relationship between clustering coefficient and technological innovation performance; that is, the wider the knowledge breadth, the flatter the U-shaped relationship between clustering coefficient and technological innovation performance	Not through
H4b	The breadth of knowledge base strengthens the positive influence of the structural hole on the technological innovation performance of enterprises	Pass
H4c	The breadth of knowledge base weakens the positive influence of network relationship strength on an enterprise’s technological innovation performance	Pass

### Research Findings

This study investigated the influence of collaborative innovation network structure and network relationship on the technological innovation performance of listed enterprises in Jiangsu, Zhejiang, and Shanghai. And this study, at the same time, analyzes the moderating effect of the enterprise’s knowledge breadth between network characteristics and technological innovation performance and draws corresponding research conclusions.

It was found that:

1. There is a U-shaped relationship between the clustering coefficient and technological innovation performance of enterprises. The main reason is that the collaborative innovation system in Jiangsu, Zhejiang, and Shanghai is still at the initial stage, which is not yet matured. Therefore, most of the innovation subjects lack cooperation experience or even cooperation consciousness. It takes a long time for each collaborative innovation subject to adapt to the changes before reaching the substantive stage of knowledge transfer
2. Structural holes positively affect the technological innovation performance of enterprises. In the collaborative innovation network, enterprises with more structural holes can have more opportunities to obtain resource heterogeneity and adjust their development strategies according to the market to improve their technological innovation performance. Therefore, enterprises with more structural holes have more advantages in technological innovation performance than the collaborative innovation network of listed enterprises in Jiangsu, Zhejiang, and Shanghai
3. The strength of network relationships positively affects the technological innovation performance of enterprises. From the perspective of trust and resource flow, a closer network relationship can bring rich emotional resources to enterprises to a certain extent, promote trust among enterprises, and be more conducive to knowledge sharing and transmission, thus improving the technological innovation performance of enterprises.
4. The knowledge breadth has no moderating effect on the relationship between clustering coefficient and technological innovation performance of enterprises. From the above analysis, it can be seen that the subjects in the cooperation network of listed enterprises in Jiangsu, Zhejiang, and Shanghai are scattered. And the degree of agglomeration is low, which is in the initial stage of building a collaborative innovation system. Enterprises lack cooperation experience and have not established a perfect trust mechanism, which leads to the fact that the breadth of their knowledge base does not play a regulatory role between them.
5. The knowledge breadth positively regulates the impact of structural holes on the technological innovation performance of enterprises. When the number of structural holes in enterprises gradually increases, the resource heterogeneity of those enterprises can also be improved. At this time, enterprises with a wide knowledge base have greater advantages in absorbing this resource heterogeneity because of their strong absorptive capacity, which is conducive to increased innovation output.
6. The knowledge breadth negatively regulates the influence of network relationship strength on the technological innovation performance of enterprises. The wider the technical fields owned by enterprises, the greater the possibility of reorganizing essential knowledge elements. Confronted with obstacles in some technical fields, enterprises can absorb relevant knowledge through collaborative innovation to make up for their shortcomings, broaden their knowledge base, and promote the improvement of technological innovation performance. However, when an enterprise tries to operate a network-wide knowledge base independently, it may lose more than it gains by choosing to strengthen cooperation intensity because it is more cumbersome for an enterprise to integrate internal innovation resources owing to extensive technical fields.

## Discussion and Conclusion

### Discussion

Extant studies on collaborative innovation networks and firms’ innovation performance have unilaterally focused on network structure or network relationships (Anqi and Shengxu, 2020; Wang and Sun, 2021). This study fills this gap by exploring the effects of collaborative innovation network structure and network relationships on technological innovation performance of listed enterprises in Jiangsu, Zhejiang, and Shanghai. In addition, this study analyzes the moderating role of enterprise’s knowledge breadth between network characteristics and technological innovation performance and draws corresponding conclusions.

Zhang et al. (2020) showed a positive relationship between clustering coefficient and focal firm innovation performance, and most studies consider a linear relationship between them (Hui and Xiaomin, 2017). However, it is arguably a bit different from our findings. Our study further suggests that a U-shaped relationship between clustering coefficient and firm technology innovation performance exists. The reason lies in that their research results can only focus on the static network. However, the corporate-related collaborative innovation networks are evolving dynamically, which is different from the relationships between clustering coefficient and technological innovation performance in different periods.

Furthermore, Wang et al. (2019) found that network relationship strength positively enhances firms’ innovation performance. Our study held that the breadth of knowledge base negatively regulates the influence of network relationship strength on the technological innovation performance of enterprises. So it is consistent with the findings of Speldekamp et al. (2020).

Finally, Yingying et al. (2020) explored the influencing factors of enterprises’ collaborative innovation under the background of COVID-19 by constructing an evolutionary game model. The research showed that the number of cooperation had a positive impact on promoting open innovation of enterprises, consistent with the research conclusions. The enhancement of network relationship strength consolidates the trust between enterprises and further promotes long-term cooperation, conducive to the steady improvement of enterprises’ technological innovation performance.

### Practical Implementations

The findings of this study bring forth practical implications. Managers can rely on internal firm resources and external networks, and institutional conditions to enhance the network collaboration efforts of their companies. Through the in-depth analysis of this study, the following vital enlightenments are obtained:

1. At the formation stage of a collaborative innovation network, enterprises participating in the network with a high degree of agglomeration will not find it conducive to their innovation because the network with a high degree of agglomeration makes the relationship between subjects more complicated and it is not easy to establish a trust relationship. Therefore, presently listed enterprises in Jiangsu, Zhejiang, and Shanghai should embed into collaborative innovation networks with moderate aggregation. Networks with different aggregation levels have different resource circulation states. Enterprises should avoid embedding networks with excessive aggregation and redundant information to integrate their resources and improve their technological innovation performance
2. From the perspective of collaborative innovation in response to the COVID-19 pandemic, the successful advancement of industry-university-research cooperation requires the guidance of the governments of the respective countries and support of the outside world. Local governments should actively build a platform for industry-university-research cooperation conducive to local economic development based on their industrial characteristics. They can also cooperate with universities to build a research base and conduct demand research and demand docking in the base, to establish a long-term cooperative relationship, which will not only improve the technological innovation performance of enterprises but also effectively improve the conversion rate of scientific and technological achievements of universities, and achieve win-win cooperation finally
3. During the pandemic, many listed companies helped fight the COVID-19. The social value generated was much higher than the economic value, which brought an excellent reputation to these companies and laid a solid foundation for long-term development. Such companies are often more responsible and should be preferred partners. Therefore, it is suggested that innovation subjects should choose responsible and trusted R&D partners in collaborative innovation. It is important for enterprises to seize the opportunity of collaborative innovation, deepen cooperative relations, gradually accumulate cooperation experience in cooperation, enhance mutual trust, establish long-term cooperation plans or build strategic alliances, and enhance cooperation ability and technological innovation performance
4. Enterprises should pay more attention to their knowledge base from the strategic level and choose to embed them into the appropriate collaborative innovation network according to the characteristics of their knowledge base. According to this study, the breadth of knowledge base significantly enhances the positive impact of structural holes on technological innovation performance, but it inhibits the positive relationship between network relationship strength and technological innovation performance. When an enterprise has a wide knowledge base, its ability to perceive and utilize innovative resources is vital. However, enterprises with a wide knowledge base often have scattered innovation resources. Too much attention to the intensity of cooperation with external subjects is not conducive to resource concentration. It consumes too much manpower and material capital, which invariably hinders new technological innovation. It will be more beneficial for enterprises to choose key areas to deepen cooperation with external bodies and pay more attention to integrating and developing internal resources in other fields. Therefore, with the rapid change of technology, it is easy for enterprises to ignore the changes in the surrounding technical environment if they only focus on the technical fields that they have specialized in. Through knowledge sharing and technical cooperation with the outside, enterprises can acquire new knowledge to maintain technical sensitivity, which positively impacts the fundamental innovation of enterprises. To enable enterprises to carry out external cooperation more effectively, enterprise managers must first check their knowledge base and determine its nature. When choosing external technical cooperation, they can fully consider the complementary degree of their original knowledge elements.

Secondly, managers should adjust their knowledge integration mechanism and strive to cultivate their knowledge integration ability to better adapt to the existing knowledge base of enterprises. To maximize the benefits brought by accumulated knowledge resources and strengthen innovation, enterprises with a vast knowledge base should strengthen their knowledge-sharing process and procedures. Enterprises with a deep knowledge base should make joint efforts to establish and improve the knowledge integration mechanism.

## Conclusion

This study takes A-share listed companies in Jiangsu, Zhejiang, and Shanghai as the research objects, constructs a theoretical model based on the two dimensions of network structure and network relationship, and constructs an empirical analysis on the relationship between the characteristics of a collaborative innovation network and the technological innovation performance of enterprises, and tests the moderating effect of the knowledge breadth between them.

The study found that the dominant position of enterprises in the collaborative innovation network can promote technological innovation performance. For example, the increase in the number of structural holes and the strengthening of network relationships improves the collaboration strength of enterprises. Simultaneously, the breadth of enterprise knowledge base plays a critical role between network characteristics and technological innovation performance. Enterprises need to choose suitable network partners according to the features of the knowledge base.

Finally, it is worth mentioning that in response to the COVID-19, the researchers find that cooperative innovation has become an inevitable trend.

The contributions of this study lie in: (1) Measuring the strength of network relations with statistical data, which makes up for the defect that questionnaire data is subjective. (2) Using DEA to measure technological innovation performance is more objective than using patent quantity to measure technological innovation performance. (3) From the regional level, the relationship between the network and technological innovation performance is studied, and the key findings are universal. They can be applied in many fields instead of specific industries.

## Limitation of the Study

The researchers identified the following study limitations: In the dimension of network relationship, only the strength of the network relationship is measured, and the indicators, such as network reciprocity, are not included. As for the adjustment variables, only the adjustment effect of knowledge breadth is verified, but the knowledge depth is not explored. Future studies would be conducted to increase the complexity of the model and look for other possible regulatory variables or intermediary variables. Secondly, only listed companies are selected in this study to ensure data availability, while other small- and medium-sized enterprises were not considered. Further research would consider expanding the scope of research objects.

## Data Availability Statement

The raw data supporting the conclusions of this article will be made available by the authors, without undue reservation.

## Author Contributions

MZ contributed to the conception of the study. SJ performed the data analyses and wrote the manuscript. ZB helped perform the analysis with constructive discussions. XH helped collect data. All authors contributed to the article and approved the submitted version.

## Funding

This study is supported by the Social Science Funding Project of Jiangsu Province under Grant No. 18GLB024.

## Conflict of Interest

The authors declare that the research was conducted in the absence of any commercial or financial relationships that could be construed as a potential conflict of interest.

## Publisher’s Note

All claims expressed in this article are solely those of the authors and do not necessarily represent those of their affiliated organizations, or those of the publisher, the editors and the reviewers. Any product that may be evaluated in this article, or claim that may be made by its manufacturer, is not guaranteed or endorsed by the publisher.

## Footnotes

^1^ www.stats.gov.cn

^2^https://view.inews.qq.com/a/20201016A009EC00, 2020-10-16.

^3^ http://www.cnpat.com.cn

^4^ http://www.baiten.cn

^5^ http://www.cninfo.com.cn
